# A Mechanistic Model for Estimating Rice Photosynthetic Capacity and Stomatal Conductance from Sun-Induced Chlorophyll Fluorescence

**DOI:** 10.34133/plantphenomics.0047

**Published:** 2023-05-09

**Authors:** Hao Ding, Zihao Wang, Yongguang Zhang, Ji Li, Li Jia, Qiting Chen, Yanfeng Ding, Songhan Wang

**Affiliations:** ^1^ Jiangsu Collaborative Innovation Center for Modern Crop Production/Key Laboratory of Crop Physiology and Ecology in Southern China, Nanjing Agricultural University, Nanjing, China.; ^2^ International Institute for Earth System Sciences, Jiangsu Center for Collaborative Innovation in Geographical Information Resource Development and Application, Nanjing University, Nanjing, China.; ^3^ State Key Laboratory of Remote Sensing Science, Aerospace Information Research Institute, Chinese Academy of Sciences, Beijing 100101, China.

## Abstract

Enhancing the photosynthetic rate is one of the effective ways to increase rice yield, given that photosynthesis is the basis of crop productivity. At the leaf level, crops’ photosynthetic rate is mainly determined by photosynthetic functional traits including the maximum carboxylation rate (*V*_cmax_) and stomatal conductance (gs). Accurate quantification of these functional traits is important to simulate and predict the growth status of rice. In recent studies, the emerging sun-induced chlorophyll fluorescence (SIF) provides us an unprecedented opportunity to estimate crops’ photosynthetic traits, owing to its direct and mechanistic links to photosynthesis. Therefore, in this study, we proposed a practical semimechanistic model to estimate the seasonal *V*_cmax_ and gs time-series based on SIF. We firstly generated the coupling relationship between the open ratio of photosystem II (qL) and photosynthetically active radiation (PAR), then estimate the electron transport rate (ETR) based on the proposed mechanistic relationship between SIF and ETR. Finally, *V*_cmax_ and gs were estimated by linking to ETR based on the principle of evolutionary optimality and the photosynthetic pathway. Validation with field observations showed that our proposed model can estimate *V*_cmax_ and gs with high accuracy (*R*^2^ > 0.8). Compared to simple linear regression model, the proposed model could increase the accuracy of *V*_cmax_ estimates by >40%. Therefore, the proposed method effectively enhanced the estimation accuracy of crops’ functional traits, which sheds new light on developing high-throughput monitoring techniques to estimate plant functional traits, and also can improve our understating of crops’ physiological response to climate change.

## Introduction

Crops assimilate carbon dioxide (CO_2_) and generate organic matter through photosynthesis, which is the basis for formulating crop productivity and grain yield. Increasing photosynthetic rate is one of the effective strategies for enhancing crop yield, to meet the growing demand of global food due to increasing population [1–3]. In general, 2 subclasses of phenotype traits, i.e., the structural traits and functional traits, largely affect the crops’ photosynthetic rate and yield. Current crop phenomics studies mainly focused on the structural traits, such as the leaf area, crop height and biomass, etc. [4,5]. However, beyond these structural traits, the photosynthetic functional traits (e.g., the maximum carboxylation rate [*V*_cmax_], the stomatal conductance [gs], etc.) were the parameters that directly related to the foliar photosynthetic rate [6], which need accurate estimation both at the site and regional scales, in order to enhance the simulation and prediction accuracy of crop productivity and grain yield [7].

Among the plenty of functional traits, representing the kinetics of the active Rubisco enzyme [8–10], *V*_cmax_ is generally used to determine the photosynthetic rate of vegetation leaves in ecosystem models [11–14]. Therefore, *V*_cmax_ is one of the key leaf traits that determine the photosynthetic rates of crops and also their response to environmental changes [15–18]. In parallel, the foliar uptake of CO_2_ and the synchronous loss of water through transpiration were both largely affected by the stomatal conductance (gs) [19], which is also a key functional trait that directly affects the foliar photosynthetic rate. Therefore, timely and accurate estimation of these photosynthetic functional traits is of vital importance for reliable prediction of crop productivity and their feedback to climate change [16,20,21] and also can provide basic support for rice breeding [22].

In recent decades, the emerging sun-induced chlorophyll fluorescence (SIF) provides us with an exceptional way for estimating crop photosynthetic functional traits. SIF is a remitted signal that could be observed in the red and near-infrared bands when the photosynthetic activity was occurred, which therefore provides us an unprecedently opportunity to accurately estimate the photosynthetic traits of plants [23,24]. Recently, SIF has been used to estimate the photosynthetic capacity parameters, both from site-level to the regional and global scales [25–28]. These studies generally are based on the simple assumption that SIF emission is linearly related to the electron transport rate (ETR) or based on the data assimilation method lacking of direct mechanism support [28]. However, a recent study has proposed a nonlinear mechanistic relationship between SIF and ETR [29], which provides an emerging way for us to extract the photosynthetic functional traits of crop vegetation with higher accuracy.

Therefore, in this study, we aim to develop a semimechanism model for estimating the photosynthetic functional traits of rice (i.e., *V*_cmax_ and gs) based on SIF observations. To this end, firstly, the coupling relationship between the open ratio of photosystem II (qL) and photosynthetically active radiation (PAR) was constructed based on the model simulations. Then, the estimation of ETR was achieved based on the proposed nonlinear mechanistic relationship of SIF [29]. Finally, *V*_cmax_ and gs were estimated by linking to ETR based on the principle of evolutionary optimality [30] and the photosynthetic pathway [31].

## Materials and Methods

### Data

We used the field measurements from a previous study [32], including the canopy SIF data, canopy reflectance data, and the leaf *V*_cmax_ and gs observations. We collected the canopy SIF observations from a rice-paddy site, which is located at Zhenjiang City, China (31°48′N, 119°13′E). This site has a typical northern subtropical and semihumid monsoon climate. The mean annual temperature of this site was 15.2 °C. The canopy SIF spectra was measured using the FluoSpec2 system [33–34] during the whole growing season in 2017 to 2018. In this system, an QEpro spectrometer was embedded with an internal shutter to measure the canopy reflectance in the range of 730 to 780 nm with a resolution of 0.15 nm. The system was radiometrically calibrated before and after the field measurements in order to eliminate the potential signal drift. Then, the canopy SIF was retrieved using the improved FLD method [34–36]. Besides, we also obtained the canopy near-infrared reflectance of vegetation (NIRv) and the fraction of absorbed PAR absorbed by green leaves (FPAR) data from the previous study [32]. The leaf *V*_cmax_ and gs measurements during the growing season of 2017 and 2018 were also obtained for validation, which were calculated by measuring the A-Ci curve using LI-6800.

### Methods

#### General description of the semimechanistic model

**Fig. 1.**
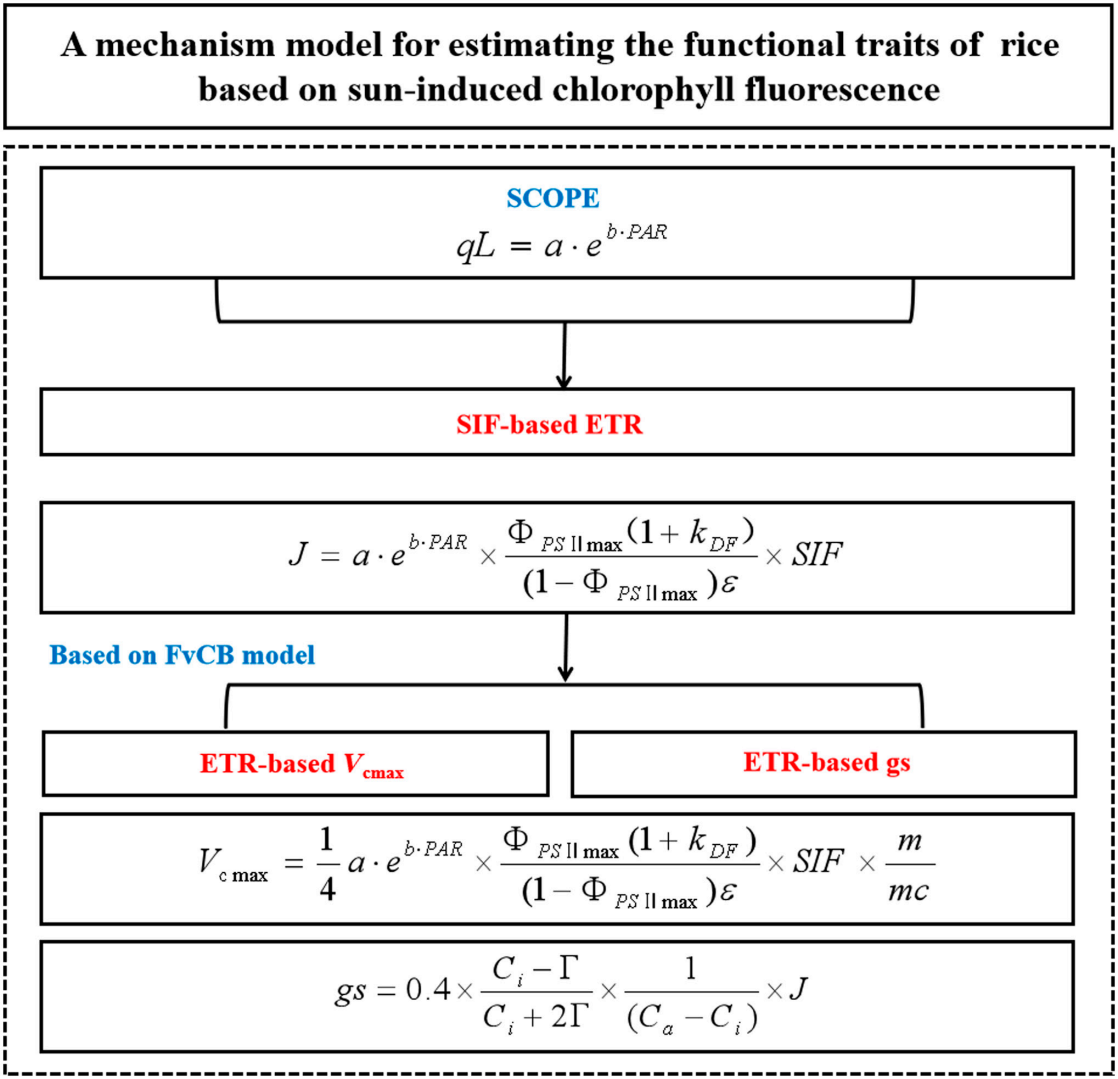
Flowchart for deriving the SIF-based *V*_cmax_ and gs. SCOPE, the Soil-Canopy-Observation of Photosynthesis and Energy fluxes model; FvCB model, the Farquhar photosynthesis model; qL, the fraction of open PSII reaction centers; PAR, the photosynthetically active radiation; ETR, the electron transport rate; FPAR, the fraction of photosynthetically active radiation; NIRv, near-infrared reflectance; SIF, sun-induced chlorophyll fluorescence; *V*_cmax_, the maximum carboxylation rate; gs, leaf stomatal conductance; Γ, the CO_2_ compensation point; *C*_a_, the ambient CO_2_ partial pressure; *C*_i_, the intercellular CO_2_ partial pressure.

The procedures for estimating *V*_cmax_ and gs from SIF were described in Fig. 1. Firstly, the empirical relationship between qL and PAR were established based on model simulations. Then, ETR was estimated from SIF based on the nonlinear mechanism relationship [29]. Finally, *V*_cmax_ was derived from ETR based on the theory of evolutionary optimality principle, and gs was estimated based on ETR through the interpretation of the photosynthetic pathway [30].

#### Generating the empirical model between qL and PAR

To derive ETR from SIF based on the nonlinear mechanism relationship [29], we need to firstly quantify the value of qL, i.e., the fraction of open PSII reaction centers. The previous study has suggested that qL generally had a nonlinear decreasing trend with the increase of PAR [29]. Therefore, we followed this finding and generated the empirical relationship between qL and PAR. This was realized through the model simulations from the Soil-Canopy-Observation of Photosynthesis and Energy fluxes model (SCOPE model) [37,38]. Based on the input parameters including leaf area index, chlorophyll content, etc., the canopy and leaf total emitted SIF signals were simulated through the SCOPE model at various conditions of PAR values (0 to 2,000 W m^−2^). Then, the qL values were calculated and the empirical relationship between qL and PAR is quantitatively fitted using an exponential function:

$$qL=a\cdot e^{b\cdot \mathrm{PAR}}$$ (1)

where qL is the fraction of open PSII reaction centers and PAR is the photosynthetically active radiation (W m^−2^); *a* and *b* were the fitted parameters using the least squares fitting method.

#### Estimating ETR from SIF

ETR can be estimated through the nonlinear relationship with SIF, based on the following equation [29]:

$$\mathrm{ETR}=qL\frac{\Phi_{\mathrm{PS}\;\mathrm{II}\;\max}\left(1+k_{\mathrm{DF}}\right)}{\left(1-\Phi_{\mathrm{PS}\;\mathrm{II}\;\max}\right)\varepsilon }\times \mathrm{SIF}$$ (2)

where Φ_PSIImax_ is the maximum photochemical quantum yield of PSII; *k*_DF_ = *k*_D_/*k*_F_, *k*_D_ is the first-order rate constants for representing the de-excitation of excited chlorophylls through the constitutive thermal dissipation, while *k*_F_ depicts the similar value via the fluorescence emissions; ε is the canopy escape probability of SIF, which could be estimated based on [39]:

$$\varepsilon \approx \frac{\mathrm{NIR}v}{\text{FPAR}}$$ (3)

where NIRv and FPAR are the reflectance in near-infrared wavelength and the fraction of PAR, respectively.

Accordingly, combining Eqs. 2 and 3, ETR could be estimated from SIF using the following equation:

$$\begin{array}{c} \mathrm{ETR}=a\cdot e^{b\cdot \mathrm{PAR}}\times \frac{\Phi_{\mathrm{PS}\;\mathrm{II}\;\max}\left(1+k_{\mathrm{DF}}\right)\text{FPAR}}{\left(1-\Phi_{\mathrm{PS}\;\mathrm{II}\;\max}\right)\text{NIRv}}\times \mathrm{SIF} \end{array}$$ (4)

where ETR is the electron transport rate (μmol m^−2^ s^−1^) and SIF is the sun-induced chlorophyll fluorescence (mW m^−2^ nm^−1^ sr^−1^).

#### Estimating *V*_cmax_ from ETR

After estimating ETR from SIF, we could estimate *V*_cmax_ from ETR by using the principle of evolutionary optimality [30]. Based on the evolutionary optimality theory that assumes plants will optimally coordinate the allocation of resources to the 2 processes of photosynthesis (the electron-transport limited and the Rubisco-limited) under normal conditions [40]:

$$A_{c}=A_{j}$$ (5)

where *A*_c_ (μmol m^−2^ s^−1^) represents the Rubisco-limited photosynthetic rate, while *A*_j_ (μmol m^−2^ s^−1^) represents the photosynthetic rate that is limited by the ETR for the regeneration of ribulose-1,5, bisphosphate:

$$A_{c}=V_{c\max}m_{c}$$ (6)

$$A_{j}=\left(\frac{\mathrm{ETR}}{4}\right)m$$ (7)

Combining Eqs. 5 to 7 and replacing ETR with Eq. 4, *V*_cmax_ was linked to SIF and expressed as:

$$\begin{array}{c} V_{c\;\max}=a\cdot e^{b\cdot \mathrm{PAR}}\times \frac{\Phi_{\mathrm{PS}\;\mathrm{II}\;\max}\left(1+k_{\mathrm{DF}}\right)\text{FPAR}}{\left(1-\Phi_{\mathrm{PS}\;\mathrm{II}\;\max}\right)\text{NIRv}}\times \mathrm{SIF}\times \frac{m}{m_{c}} \end{array}$$ (8)

$$m_{c}=\frac{C_{i}-\Gamma }{C_{i}+K_{m}}$$ (9)

$$m=\frac{C_{i}-\Gamma }{C_{i}+2\Gamma }$$ (10)

where *C*_i_ is the intercellular CO_2_ partial pressure, Γ is the CO_2_ compensation point, *K*_m_ is a relatively stable constant.

#### Estimating gs from SIF

At the leaf level, gs can be mechanistically linked to ETR based on the photosynthetic pathway. Based on Eq. 4, SIF was highly correlated with ETR [41]. Then, the gs could be estimated from SIF by using ETR as an intermediary [42–44]. The detailed procedures were presented below.

The net photosynthetic rate (*A*) could be represented as the product of photosynthetic pathway and the electron-use efficiency (EUE) [31]:

A=ETR×EUE (11)

where EUE is electron-use efficiency. The EUE for C_3_ and C_4_ plants were different, depending on the kinetic control of stoichiometry by temperature and CO_2_ concentration [12,45]. For rice, a typical C_3_ plant, the EUE can be expressed as:

$$\mathrm{EUE}=\frac{1}{4}\times \frac{C_{i}-\Gamma }{C_{i}+2\Gamma }$$ (12)

Combining Eqs. 11 and 12:

$$A=\frac{\mathrm{ETR}}{4}\times \frac{C_{i}-\Gamma }{C_{i}+2\Gamma }$$ (13)

Additionally, *A* can also be expressed as the functions of gs [19]:

$$A=\mathrm{gs}\cdot \frac{C_{a}-C_{i}}{P}$$ (14)

where *C*_a_ is the ambient CO_2_ partial pressure, *P* is the total air pressure (hPa) and was set to be 1.6 in this study [31]. After that, we get:

$$\mathrm{gs}=\frac{1.6A}{\left(C_{a}-C_{i}\right)}$$ (15)

Combining Eqs. 13 and 15:

$$\mathrm{gs}=0.4\times \frac{C_{i}-\Gamma }{C_{i}+2\Gamma }\times \frac{1}{\left(C_{a}-C_{i}\right)}\times \mathrm{ETR}$$ (16)

Replacing ETR in Eq. 4 with Eq. 16, then we can estimate gs from the SIF observations:

$$\begin{array}{c} \begin{array}{c} \mathrm{gs}\;=\;0.4\times \frac{C_{i}-\Gamma }{C_{i}+2\Gamma }\times \frac{1}{\left(C_{a}-C_{i}\right)}\times a\cdot e^{b\cdot \mathrm{PAR}}\\ \times \frac{\Phi_{\mathrm{PS}\;\mathrm{II}\;\max}\left(1+k_{\mathrm{DF}}\right)\text{FPAR}}{\left(1-\Phi_{\mathrm{PS}\;\mathrm{II}\;\max}\right)\text{NIRv}}\times \mathrm{SIF} \end{array} \end{array}$$ (17)

#### Statistical analyses

We firstly obtained the field SIF, *V*_cmax_ and gs time-series during the whole rice growing season in 2017 to 2018. Then, based the established semimechanistic models above, we estimated *V*_cmax_ and gs from the field SIF observations and compared them with the field observations. The accuracy of the models was represented by indices, including the determination coefficient (*R*^2^), the mean absolute error (MAE) and the root-mean-squared error (RMSE). We used the MATLAB2020 to perform all of the statistical analyses in this study.

Besides, we also conducted the sensitivity analysis of our models to the parameters. The estimation of *V*_cmax_ and gs from SIF requires some parameters, mainly including *k*_DF_ and Φ_PSIImax_ [29]. *k*_DF_ was generally presumably a constant scalar, because *k*_D_ and *k*_F_ are often considered as intrinsic physical properties of chlorophyll molecules and treated as constants [46]. Here in this study, following the previous study, we take *k*_DF_ as a value of 19 [29]. Furthermore, although Φ_PSIImax_ could be affected by photoinhibition, it is generally highly converged for a certain type of plant under unstressed conditions. Therefore, in this analysis, following the previous study [29], we take a value of Φ_PSIImax_ to be 0.83. Although *k*_DF_ and Φ_PSIImax_ are expected to be stable under unstressed conditions for rice systems, the potential impacts of their variations need to be explored so that the SIF-based model of *V*_cmax_ and gs estimations can be applied to a broad range of environmental conditions. Therefore, we conducted an uncertainty analysis by giving a 10% changes of the original Φ_PSIImax_ and *k*_DF_ values, and calculated the *V*_cmax_ and gs again. The impacts of the parameters changes on the models were then analyzed.

## Results

### ETR estimations from SIF

**Fig. 2.**
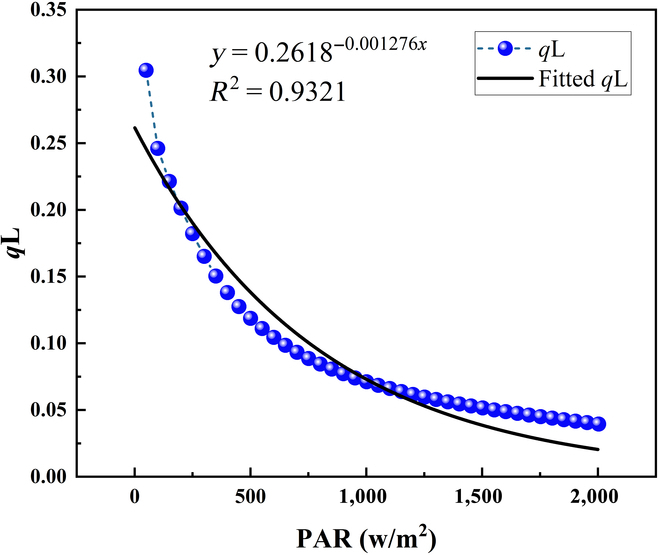
The fitted relationship between qL and PAR. Blue circles indicate the simulated qL and PAR data points. The black line represents the fitted lines.

We first generated the coupling relationship between qL and PAR in Eq. 1 based on the exponential function. The qL variations for rice cropping system under various illumination conditions were simulated based on the SCOPE model [37,38]. Overall, 40 pairs of qL and PAR data points were used (Fig. 2). In general, a reduction of the qL value was found with the increases of PAR, but their relationship is nonlinear. We then used the exponential function to fit the relationship between qL and PAR, which generally had a good performance with the *R*^2^ value of 0.93.

**Fig. 3.**
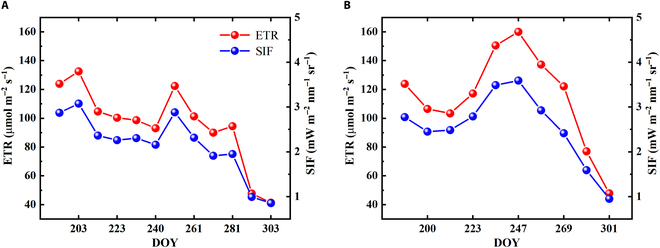
The estimated ETR based on SIF observations during 2017 (A) and 2018 (B). The red and blue dots represent the estimated ETR and SIF observations, respectively.

Then, based on Eq. 4, the ETR during the rice growing seasons of 2017 and 2018 was calculated. In general, the seasonal variations of canopy SIF and ETR were the similar (Fig. 3). Both the canopy SIF and the estimated ETR showed relatively higher values during the first half than the second half of growing seasons. Additionally, a prominent consistency of the highest point for SIF and ETR was also found at DOY (day of year) 203 in 2017 and DOY 247 in 2018. These results indicated a strong correlation between SIF and ETR during the entire growing season and indicated that our model would be useful for predicting ETR across seasonal scales.

### The performance for estimating *V*_cmax_ from SIF

**Fig. 4.**
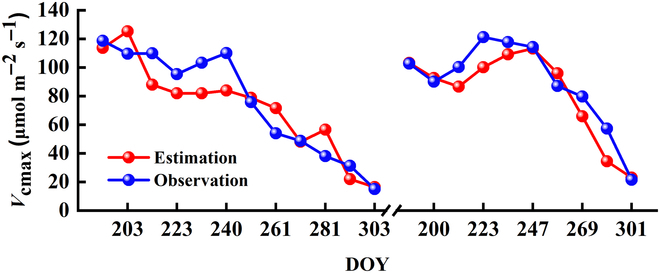
The estimated *V*_cmax_ based SIF observations during 2017 and 2018. The red and blue dots represent the estimated *V*_cmax_ and field *V*_cmax_ observations, respectively.

We then assessed the model’s performance of estimating *V*_cmax_ from SIF observations. The observed and estimated *V*_cmax_ for during the whole growing seasons of 2017 and 2018 were presented in Fig. 4. The seasonal dynamics of the observed and estimated *V*_cmax_ is generally consistent and agreed well with each other. *V*_cmax_ generally showed a decreased trend with the increase of DOY, which is both found from the modeled estimations and field observations (Fig. 4). The estimated *V*_cmax_ showed a peak value of around 130 μmol m^−2^ s^−1^ at the start of the growing season during 2017, while the observed *V*_cmax_ also showed a peak of around 120 μmol m^−2^ s^−1^ at the early stage of growing season (Fig. 4). Nevertheless, the estimated *V*_cmax_ showed a peak value of about 110 μmol m^−2^ s^−1^ during the middle of the growing season of 2018, which is not observed during 2017. This phenomenon may be resulted from the increased temperature during around DOY 230, which led to the increased activity of Rubisco enzyme, and thus accelerated the carboxylation rate and increased *V*_cmax_.

**Fig. 5.**
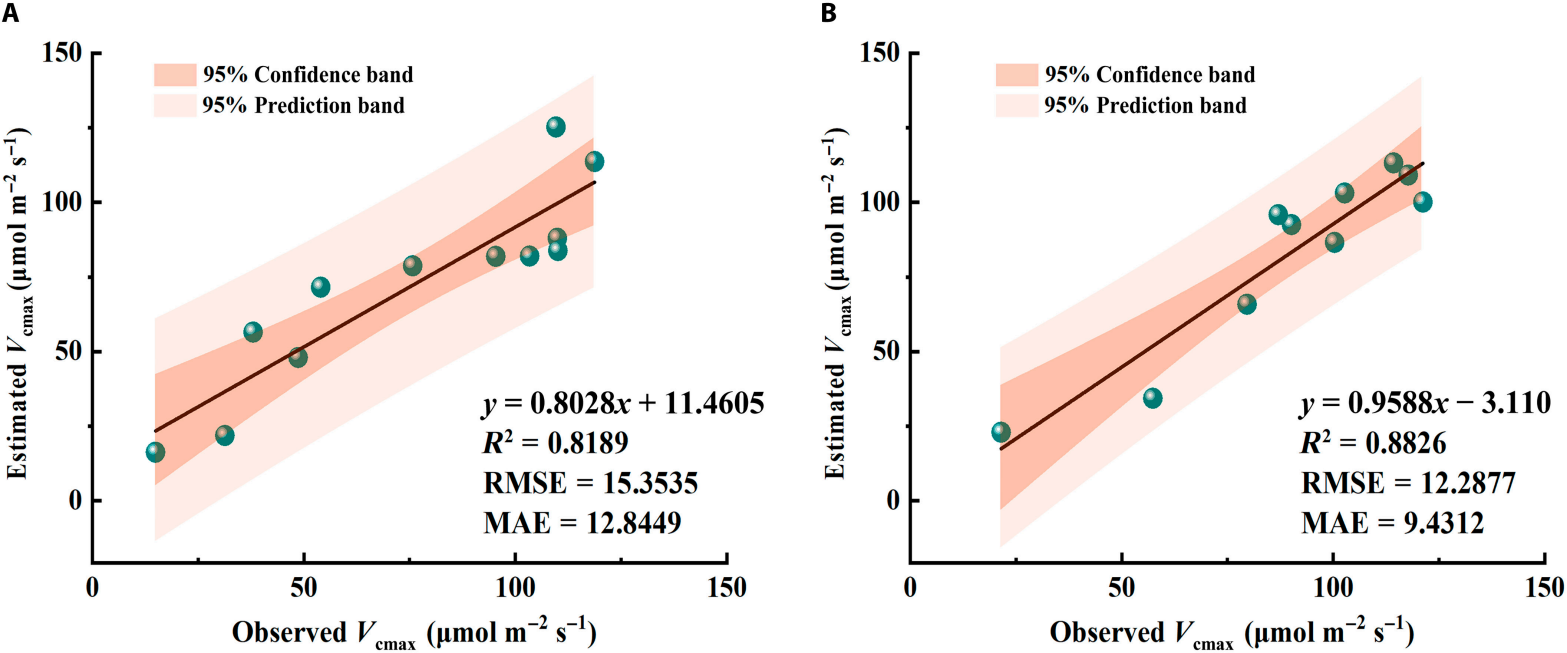
The scatter plot of the estimation results of *V*_cmax_ of 2017 (A) and 2018 (B). The black lines represent the fitting relationship between estimated and observed *V*_cmax_.

The correlations between the modeled and observed *V*_cmax_ were then analyzed. We found that the modeled *V*_cmax_ could capture more than 80% of the *V*_cmax_ variations both for 2017 and 2018, with an *R*^2^ value of 0.82 and 0.88, respectively (Fig. 5). The slopes of the regression lines between the modeled and observed *V*_cmax_ were near 1 (0.80 and 0.96, respectively), which suggested that the proposed model could reproduce the observed *V*_cmax_ with almost no biases. Moreover, the proposed model generally had high accuracy for estimating *V*_cmax_, with the RMSE values of 15.35 and 12.29 μmol m^−2^ s^−1^ for 2017 and 2018, respectively. These results above indicated the general good performance of our proposed model for estimating *V*_cmax_ based on the field SIF observations.

### The performance for estimating gs from ETR

**Fig. 6.**
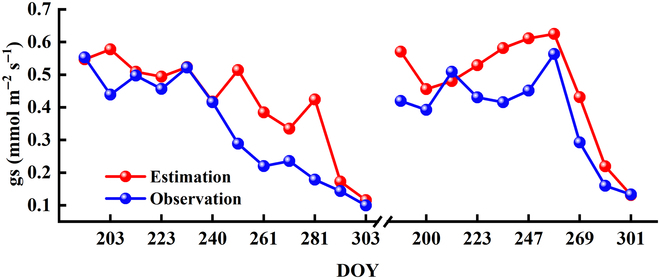
The estimated gs based on SIF observations during the growing seasons of 2017 and 2018. The red and blue dots represent the estimated gs and field gs observations, respectively.

Based on the proposed models and the field SIF observations, we estimated the gs of rice leaves during 2017 and 2018 (Fig. 6). Similar to the results of *V*_cmax_, the seasonal trajectories of estimated gs and field gs observations were consistent. In general, a clear decrease trend of gs in 2017 was found with the increase of DOY, and the peak of gs occurred at the early stage of growing seasons. However, the gs in 2018 had a slightly increase during the early growing season before DOY 253 and had a sharp decrease at the second half of the growing season (Fig. 6).

**Fig. 7.**
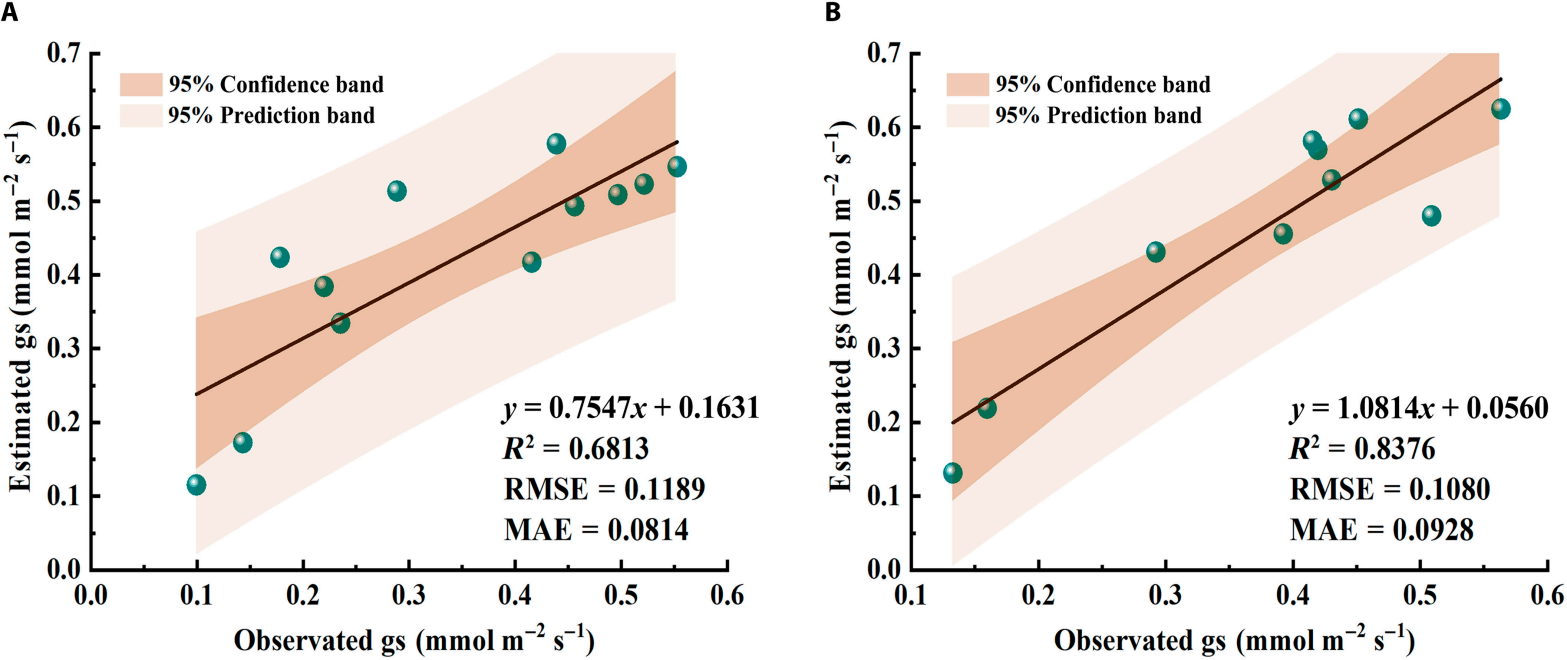
The scatter plot of the estimation results of gs of 2017 (A) and 2018 (B). The black lines represent the fitting relationship between estimated and observed gs.

More importantly, a direct comparison of modeled gs and the field observations was shown in Fig. 7. The *R*^2^ between estimated and observed gs were 0.68 and 0.84 for 2017 and 2018, respectively. For 2017, our model had a slight overestimation of the gs, given that the slope of the linear regression was smaller than 1 (i.e., 0.75, Fig. 7A). This overestimation was largely presented at the late stage of the growing seasons, i.e., after DOY 240 (Fig. 6A). For 2018, both the *R*^2^ and the RMSE values showed that the accuracy of our proposed model was higher than that for 2017 (Fig. 7). In summary, our proposed model offered a simple and accurate method for estimating gs from SIF observations, which was based on the nonlinear relationship between SIF and ETR.

## Discussion

### Advantages of the proposed model

Recently, SIF signals have emerged as a promising tool to estimate the vegetation gross primary production at foliar, canopy, and global scales, given that SIF emissions were directly linked to vegetation photosynthesis. Also, based on the advantages of SIF for indicating plant photosynthetic activity, several attempts have been conducted to estimate the photosynthetic parameters (including *V*_cmax_ and gs) from SIF [25–26,28]. These studies generally relied on the basis that SIF is linked to the electron transport process during the photosynthetic activities at the foliar level [42,47]. However, these studies were generally based on the simple linear relationship between SIF and ETR, or through the data assimilation approach that relied on the linear relationship between SIF and gross primary production [28,43], but largely ignored the nonlinear mechanistic relationship between SIF and ETR at the foliar scale [29]. Due to the rapidly growing observations of SIF both at the ground sites and satellite platforms [23,48–50], a practical mechanistic method to estimating photosynthetic parameters from SIF is needed.

Therefore, this study proposed a semimechanism model for estimating the photosynthetic functional traits of rice, including *V*_cmax_ and gs, based on field SIF observations. Validation results based on field observations showed the high accuracy of our model for estimating *V*_cmax_ and gs. The framework of our model differs from previous statistical models, due to our derivation is based on the nonlinear mechanistic relationships between ETR and SIF [29]. Moreover, we have also derived the empirical relationship between qL and PAR for rice cropping system and made the accurate estimation of ETR, *V*_cmax_, and gs from SIF reliable. Compared to the estimation method that using the simple empirical relationship between SIF and *V*_cmax_, our proposed model had a better accuracy. Compared the estimated *V*_cmax_ to field observations, the RMSE value for our proposed model was solely 14.04 μmol m^−2^ s^−1^, while the RMSE value for the simple linear regression method was 22.32 μmol m^−2^ s^−1^ (Table).

**Table.** The accuracy of the *V*_cmax_ estimations between different methods. “Linear” represents that using the simple empirical relationship between SIF and *V*_cmax_. “Nonlinear” represents that using the proposed method in this study.

Methods	*R* ^2^	Mean absolute error (MAE, μmol m^−2^ s^−1^)	Root mean square error (RMSE, μmol m^−2^ s^−1^)	Accuracy improvement (%)
Linear	0.55	19.02	22.32	40.6
Nonlinear	0.84	11.29	14.04	

**Fig. 8.**
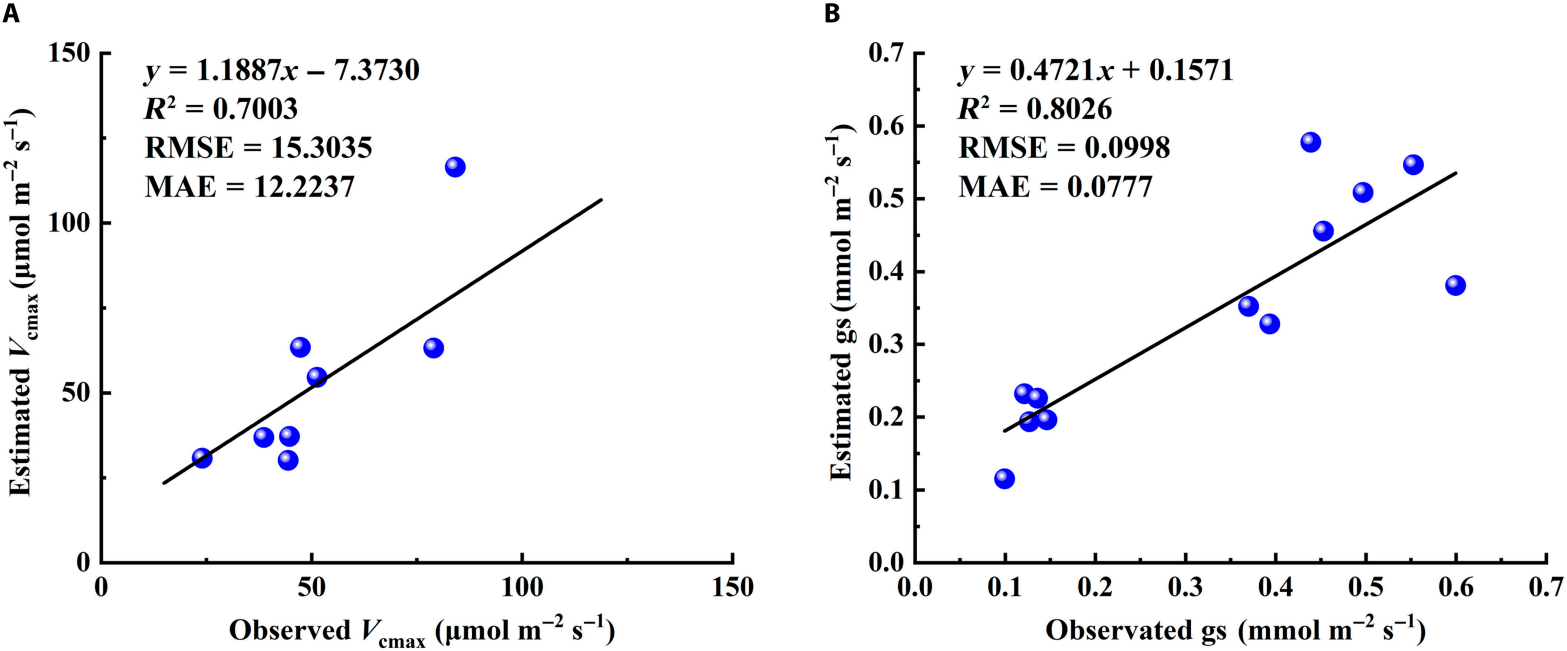
The scatter plot of the estimation results of *V*_cmax_ (A) and gs (B) at Danyang station. The black lines represent the fitting relationship between the estimated and the observed *V*_cmax_ and gs.

In addition, we used the field observations from another station to further verify the robustness of our model. The data were acquired from the Danyang station during 2022, which is located at Zhenjiang City, China (31°54′N, 119°28′E). The photosynthetic functional traits (*V*_cmax_ and gs) of rice during the whole growth period were observed, and we then verified our model based on these observations. Figure 8 shows the correlation analysis between observed and estimated *V*_cmax_ and gs, respectively. The *R*^2^ between estimated and observed *V*_cmax_ and gs were 0.70 and 0.80, respectively (Fig. 8). In conclusion, the accuracy of our semimechanistic model was also verified at the Danyang site, which showed that the model performed well and could be used to effectively estimate the photosynthetic functional traits of rice at different sites.

**Fig. 9.**
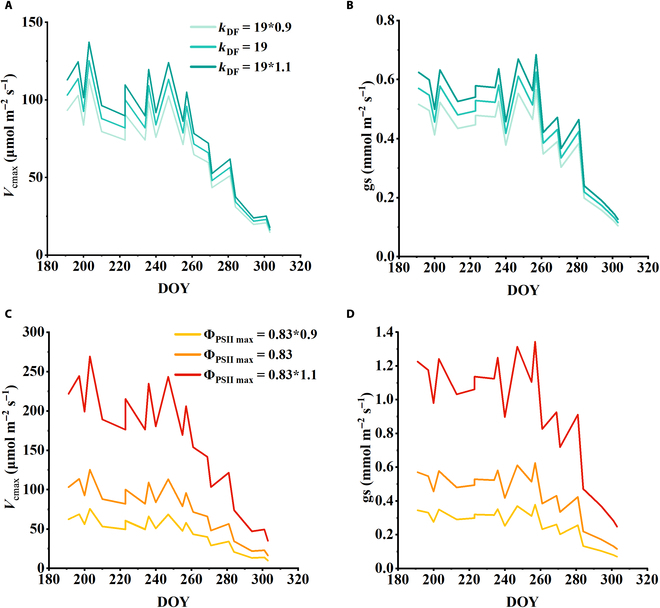
Sensitivity of *V*_cmax_ and gs estimations to *k*_DF_ and Φ_PSIImax_. (A and B) Sensitivity of *V*_cmax_ estimations to the changes of Φ_PSIImax_ and *k*_DF_. (C and D) Sensitivity of gs estimations to the changes of Φ_PSIImax_ and *k*_DF_.

### Uncertainty in the proposed model

Although our proposed model can estimate the functional traits of rice with a high accuracy, some uncertainties still lie ahead. Firstly, using this model needs some parameters, such as the qL, *k*_DF_, Φ_PSIImax_, and ε. First, qL had to be estimated before the model can be applied widely. qL was less observed and studied than some other photosynthetic parameters and has not been generally reported in leaf-level measurements. However, our derivation of the empirical relationship between qL and PAR make the predictions of qL at different light conditions reliable. Although the relationship between qL and PAR is empirical and is generated from the model simulations, it is generally consistent with the field observations from a recent study [51]. Based on the pulse amplitude modulated measurements, an exponential formulation between the qL and PAR for C_3_ crops was derived [51], which was consistent with our empirical equation derived from the model simulations. This further verified the robustness of our results. Nevertheless, there is still a need for measurements that allow for developing and testing a predictive model of qL as a function of various environmental conditions and canopy depth. In addition to qL, better understanding and prediction of ε are also crucial for the application of SIF–ETR relationship. Though it is possible to model ε from the simple method closely related to FPAR and NIRv, i.e., based on the canopy radiative transfer principles [39,52], direct measurements are still needed to verify its performance.

In addition to the qL and ε, K_DF,_ and Φ_PSIImax_ are also important parameters of our proposed model, which are generally suggested to be related to vegetation types and environmental conditions. Following the former study [29], we take these 2 parameters using the following values: *k*_DF_ = 19, Φ_PSIImax_ = 0.83. These values are chosen for their representativeness for typical C_3_ vegetation at unstressed conditions [39,47,53]. As suggested by a previous study [29], K_DF_ and Φ_PSIImax_ were generally highly converged for a certain type of plant under normal climate conditions, which is feasible for this study that only aimed at rice paddy. However, in order to extend this method to a wider range of crop types, we still need to investigate the uncertainties of the model with the changes of these 2 parameters. Therefore, by setting a ±10% changes for *k*_DF_ and Φ_PSIImax_, respectively, and keep the other parameters stable, we calculated the sensitivity of estimation results of *V*_cmax_ and gs to those 2 parameters (Fig. 9). With the changes of both *k*_DF_ and Φ_PSIImax_, the seasonal variations of *V*_cmax_ and gs were generally the similar. However, a great sensitivity of *V*_cmax_ and gs estimations to Φ_PSIImax_ was found. In comparison, the sensitivity of *V*_cmax_ and gs estimates to *k*_DF_ was smaller. These results suggested that when using the proposed model in this study, the setting of Φ_PSIImax_ need to be accurate.

### Implications and outlook

Our model has important implications for estimating the functional traits of vegetation based on SIF observations. In this study, an existing framework for modeling ETR, *V*_cmax_ and gs were proposed, providing an improved semimechanistic model for accuracy estimation of rice functional traits. Our method can easily be extended to other crops, which is beneficial to the accurate assessment of global crop photosynthesis and accurate prediction of crop yield, and provides a basis for the assessment of global carbon cycle. Also, high-throughput monitoring and analysis of photosynthetic rate and photosynthetic phenotype parameters of crops can be realized.

Moreover, extending this model from the site level to regional and global scales, the proposed model can estimate ETR, *V*_cmax_, and gs at different scales using the satellite SIF observations. The derived ETR and *V*_cmax_ were both the key parameters of vegetation photosynthesis processes. The accurate estimations of these parameters could effectively improve the performance for modeling crop carbon and water fluxes at large scales and finally lead to the higher accuracy of crop yield predictions [54]. The link between SIF and gs for C_3_ plants was also established in this study, which provides an improved method to estimate gs for various C_3_ crops [31] and is also expected to enhance our understandings of how the stomata of crops response to global climate change. In addition, based on the developed model in this study, we could then estimate the vegetation transpiration through the mechanistic relationship between transpiration and gs [31]. The regional and global ecosystem evapotranspiration could also be finally derived based on satellite SIF data. Besides, by expanding this model into larger spatial scale based on satellite SIF [55], we could estimate the rice functional traits, including ETR, *V*_cmax_, gs, and transpiration at the regional and global scales.

Nevertheless, there are still some caveats needed to be improved in the future. First, accurately observing SIF remains a challenging task, and there is an urgent need to upgrade the data quality [29,34,56]. The relationships between SIF and ETR in current study are based on some assumptions [29], may also produce uncertainties at the canopy scales. For example, the excitation capture efficiency of the open PSII centers may change under stressed climate conditions in order to avoid permanent photodamage, which possibly affects the derived relationship between SIF and ETR [29,57]. On this aspect, the relationships between SIF and ETR at various environmental conditions including the stressed environments need further investigation in the future. Besides, the SIF–gs relationship is complex with changing environment and potential collinear variations in multiple parameters, which also calls for further experiments and works.

### Conclusion

In summary, this study presents a semimechanistic model to estimate the seasonal *V*_cmax_ and gs time series based on the field SIF observations. We firstly derived an empirical relationship between the qL and PAR, incorporated this relationship into the nonlinear mechanistic relationship between ETR and SIF, and finally derived a simple and feasible model for estimating ETR from SIF observations. In the next steps, based on the ETR estimations, *V*_cmax_ and gs of rice cropping system were estimated by linking to ETR based on the principle of evolutionary optimality and the photosynthetic pathway, respectively. The estimated *V*_cmax_ and gs from our proposed model generally had excellent agreement with the field observations, in general with the *R*^2^ values higher than 0.8. Moreover, compared to previous models using the simple linear regressions between SIF and *V*_cmax_, our proposed model in this study can largely increase the accuracy of the estimations of *V*_cmax_, with the reductions of the RMSE values of >40%. Therefore, the proposed model in this study provides us an applicable method to estimate the crop functional traits with high accuracy, which provide new insights of how crop physiology response to climate change from site to regional scales, and could also be beneficial to the plant phenomics studies relating to crop functional traits.

## Data Availability

The data of this study is available from the corresponding authors upon reasonable request.
